# hsa_circ_0002872 functions in lung cancer prognostic and tumorigenesis through regulating ZBTB46 via sponging hsa-miR-29b-1-5p

**DOI:** 10.1016/j.clinsp.2025.100733

**Published:** 2025-08-07

**Authors:** Xifan Wang, Xiaoyan Song, Hongxuan Wei, Xiaoqing Li, Huihui Li, Yan Zhu, Chuandong Zhu, Fangfang Chen

**Affiliations:** a Department of Oncology, The Second Hospital of Nanjing, Affiliated to Nanjing University of Chinese Medicine, Nanjing 210003, Jiangsu, China; b Institute of Laboratory Medicine, Jinling Hospital, Affiliated Hospital of Medical School, Nanjing University, Nanjing 210002, Jiangsu, China; c The First School of Clinical Medicine, Southern Medical University, Guangzhou, China

**Keywords:** Lung Cancer, hsa_circ_0002872, hsa-miR-29b-1-5p, ZBTB46, Immune

## Abstract

**Background:**

Lung cancer is a leading cause of cancer deaths worldwide, underscoring the need for new biomarkers. Circular RNAs (circRNAs) exhibit potential as biomarkers for lung cancer. ZBTB46 is linked to lung cancer prognosis. This study intends to investigate circRNA expression and the role of ZBTB46 in predicting and treating lung cancer.

**Methods:**

CircRNAs demonstrate differential expression in lung cancer and paracarcinoma tissues, as identified by high-throughput sequencing (RNA-seq). DEcircRNAs were analyzed through GO and KEGG analyses. A potential circRNA-miRNA-mRNA axis was predicted based on ceRNA theory. ZBTB46 expression in lung cancer tissue was compared to normal tissue. Using the TCGA database, protein expression was compared through the CPTAC and HPA databases.

**Results:**

We identified 411 differentially expressed circRNAs, of which 365 were down-regulated and 46 were up-regulated. By combining qRT-PCR validation results, ceRNA theory, and intersecting data from several databases, hsa_circ_0002872/hsa-miR-29b-1–5p/ZBTB46 was predicted as the most promising downstream regulatory axis. ZBTB46 mRNA and protein expression were reduced in human lung cancer tissue compared to normal tissue.

**Conclusions:**

hsa_circ_0002872/hsa-miR-29b-1–5p/ZBTB46 represents an axis that may influence lung cancer progression, with the molecule ZBTB46 potentially inhibiting lung cancer progression and serving as a useful prognostic indicator.

## Highlights

• 411 differentially expressed circRNAs found in lung cancer and paracarcinoma tissues by high ‒ throughput sequencing.
• hsa_circ_0002872/hsa-miR-29b-1-5p/ZBTB46 axis predicted to affect lung cancer progression.
• ZBTB46 expression in lung cancer correlates with prognosis, immune cell infiltration, and immune checkpoints.
• Study suggests ZBTB46 as a potential biomarker and target for lung cancer immunotherapy.

## Introduction

Lung cancer is the leading cause of cancer-related mortality globally,^1^^,^^2^ accounting for approximately 25 % of all cancer deaths (29 % in males versus 26 % in females).^3^ Non-Small Cell Lung Cancer (NSCLC), which constitutes over 83 % of all cases, remains challenging to treat due to the limited efficacy of conventional therapies, including surgery, chemotherapy, and radiotherapy.^4^ Identifying specific biomarkers for early detection and disease monitoring is critical.

Circular RNAs (circRNAs), emerging as stable long non-coding RNAs with distinct regulatory functions, exhibit tissue specificity and modulate gene expression through miRNA sponging, transcriptional regulation, and protein interactions. CircRNAs have been found in various malignancies, including gastric, breast, and lung cancer.^5^, ^6^, ^7^ Dysregulated circRNA expression has been linked to tumorigenesis, influencing cell proliferation, migration, and differentiation.^8^^,^^9^ Mechanistically, circRNAs often act as miRNA sponges in circRNA-miRNA-mRNA regulatory networks,^10^ such as CircITGA7 inhibiting NF1 translation via miR370–3p binding.^11^ While circRNAs show promise as biomarkers for NSCLC,^12^ their underlying roles remain unclear.

ZBTB46 (also known as BTBD4, BZEL, zDC, RINZF, and ZNF340), a BTB-ZF transcription factor and well-established marker of classical Dendritic Cells (cDCs),^13^^,^^14^ has been implicated in gastric,^15^ neuroendocrine prostate,^16^ pancreatic,^17^ and colon cancer.^18^ In CRPC, ZBTB46 upregulation promotes cell proliferation through reciprocal regulation with PCK1.^16^ ZBTB46 plays a critical role in tumor immunity by governing the ontogeny and functional integrity of conventional Dendritic Cells-2 (cDC2), thus regulating antigen presentation and T-cell immunity within the tumor microenvironment.^19^ Notably, ZBTB46 has not been previously investigated in lung cancer. Despite its emerging role as a key regulator in immunity, the role of ZBTB46 in lung cancer progression, particularly regarding immune infiltration and immune checkpoint pathways, remains uncharacterized.

In the present study, we characterized the circRNA expression landscapes of lung cancer and paracancerous tissues to elucidate the functional roles and underlying mechanisms of ZBTB46 in lung cancer pathogenesis, thereby providing novel therapeutic strategies for this disease.

## Materials and methods

### Samples collection

We collected adjacent normal and cancerous tissues from patients (*n*= 15) without neoadjuvant therapy, hospitalized in the Cardiothoracic Surgery Department of Jinling Hospital from June 2018 to July 2021, and diagnosed with NSCLC. Immediately following surgery, specimens were collected and stored in liquid nitrogen. Serum samples obtained from three lung cancer patients and three healthy controls were used for high-throughput sequencing, while 15 pairs of cancer and paracancerous tissues were used for candidate circRNA qRT-PCR validation. The study was approved by the Institutional Ethics Committee of Jinling Hospital. Clinical Trials follow the CONSORT Statement rules.

### RNA extraction, circRNA library prep, and high-throughput sequencing

RNA extraction was performed using TRIzol reagent (Invitrogen) following the manufacturer's protocol. RNA integrity was evaluated via 1 % agarose gel electrophoresis to confirm high-quality samples. Only RNA meeting the quality criteria was proceeded to subsequent experimental steps. Libraries were constructed using the Illumina TruSeq RNA Library Preparation Kit (Illumina) according to the manufacturer's instructions. Key steps included:

1. Poly-A mRNA purification from total RNA using poly-T oligo-conjugated magnetic beads.
2. RNase R treatment before fragmentation to selectively degrade linear polyadenylated mRNA.
3. mRNA fragmentation via divalent cation-mediated hydrolysis at elevated temperatures.
4. First-strand cDNA synthesis with random hexamers and reverse transcriptase.
5. Second-strand cDNA generation using DNA polymerase I and RNase H.
6. cDNA end-repair and adapter ligation.
7. PCR amplification for library enrichment.

Post-PCR, amplified cDNA fragments were ligated to Illumina TruSeq adapters using the NEB ligation kit, adhering strictly to the manufacturer's protocol. This step ensures adapter compatibility with the Illumina HiSeq 4000 platform, enabling accurate high-quality sequencing. Initial QC of sequencing data was conducted, followed by STAR aligner mapping to the reference genome. CircExplorer2 was used to detect, filter, and annotate circRNAs via back-splice junction analysis.

### Bioinformatics data analysis

CIRCexplorer2 identifies and quantifies circRNAs by detecting back-splice junctions in RNA-seq data. Circprimer2 analyzes circRNA backsplice junctions to characterize circular structures. STAR aligner maps reads to the reference genome, providing alignment files for downstream analysis. Read counts were normalized using RPKM (reads per kilobase of transcript per million mapped reads) to correct for technical variations. Differentially expressed circRNAs were identified based on log2-fold change ≥ 1.2 and adjusted *p*< 0.05. Expression patterns were visualized via volcano plots and clustering. Functional annotation of host genes was performed using GO (http://geneontology.org/) and KEGG (https://www.kegg.jp/) databases, with statistical significance determined at adjusted *p*< 0.05.

### Real-time quantitative reverse transcription PCR(qRT-PCR)

GO and KEGG pathway enrichment analyses of circRNA host genes identified nine candidate circRNAs significantly implicated in lung cancer pathogenesis. The circprimer2 software was employed to determine optimal primer lengths and ensure specificity, with all primers listed in Table 1. Sequencing results were validated by qRT-PCR. Total RNA was extracted from tissues using TRIzol reagent (Invitrogen) following the manufacturer's protocol. Reverse transcription was performed using the PrimeScriptTM RT-PCR Kit (Takara, RR036A). Quantitative real-time PCR (qRT-PCR) was conducted on an ABI 7500 system using Takara SYBR Premix ExTaq Kit (RR420A). Relative expression levels of circular RNAs were calculated using the 2^-ΔΔCt^ analysis.

**Table 1:** Basic information and primers of the 9 candidate circRNAs.

CircRNA	Best transcript	GeneSymbol	F-primer	R-primer
hsa_circ_0025128	NM_001065	TNFRSF1A	CCTGCCAGGAGAAACAGAAC	GGTGAGGGACCAGTCCAATA
hsa_circ_0003922	NM_001080391	SP100	CAACAGAGTCCTGCGAACAA	GAGCAGCCTGTCATCTACACC
hsa_circ_0002872	NM_001105244	PTPRM	CCAGAACCCGAGAAACAGAC	CGAGGACGTTCTTCCTCAAC
hsa_circ_0135681	NM_014751	MTSS1	GCCAACCAGCTGGATAAAGA	CACATCCTGGTGAGAGCAGA
hsa_circ_0095155	ENST00000264033.4	CBL	ATTAACCAACCGGCACTCAC	GCAGAAGGTCAAGTCGTGGT
hsa_circ_0002470	NM_022873	IFI6	TGGTCTGCGATCCTGAATG	ACTGCAAGTGAAGAGCAGCA
hsa_circ_0046263	NM_000918	P4HB	TGGGATCACTTCCAACAGTG	TCTTCAGCCAGTTCACGATG
hsa_circ_0079440	NR_033436	PHF14	CAGACAGGCTACGGATGGAT	CAGACAGGCTACGGATGGAT
hsa_circ_0008590	NM_006509	RELB	CTGCTTCCAGGCCTCATATC	GGTCAATGCCCAGTTGAATC

### Prediction of circRNA-miRNA-mRNA axis

Two circRNAs were verified by qRT-PCR to be expressed in clinical samples consistent with those in the previous bioinformatics analysis. To predict downstream regulation of the circRNAs, these two circRNAs were validated using qRT-PCR, and miRNAs targeting these circRNAs were identified by Miranda, TargetScan, and PITA. After that, the predicted results from each database were intersected using a Venn diagram online construction site. Two DEcircRNAs targeted 2 and 5 miRNAs, respectively, and for the target miRNAs of each circRNA, candidate miRNAs of DEcircRNAs were predicted using 4 miRNA target prediction databases: miRTarBase, miRDB, TargetScan, and miRWalk. Finally, the intersection of the 4 lists was taken as the final target mRNAs. Then the authors took the intersection of mRNAs targeted by different miRNAs of the same circRNA and found that 2 targeting miRNAs of one of the circRNAs could target 11 mRNAs together. Gene expression in lung cancer was assessed through a web-based tool (GEPIA2). The authors found 1 gene that was expressed lowly in lung cancer but was strongly associated with malignancy. Based on the theory of ceRNA, the authors predicted a possible axis of low-expressed circRNA/high-expressed miRNA/low-expressed mRNA with targeted miRNAs in the ENCORI database.

### Gene data acquisition and differential expression analysis

This analysis identified ZBTB46 (ENSG00000130584), a gene not previously implicated in lung cancer. The authors obtained 1145 samples from the Cancer Genome Atlas (TCGA) database. These samples included the two major non-small cell lung cancer subtypes (LUAD and LUSC) and normal tissue samples. Specifically, the dataset comprised 106 matched cancer and paracancerous tissue pairs, 1037 cancer tissue specimens, and 2 unpaired normal tissue samples. Assays were conducted using transcripts per million kilobases. The data were normalized, and then differential expression was analyzed in *R* (version 3.6.3), and plots were generated using the ggplot2 (version 3.3.3) package. Protein differential expression was generated by the NCI/NIH Clinical Proteomic Tumor Analysis Consortium (CPTAC). The authors also explored ZBTB46 protein expression in LUAD and LUSC and normal lung tissues using the Human Protein Atlas (HPA) database.

### Multiple models on prognosis analysis

The authors investigated the correlation of the expression of candidate genes with clinical prognosis using online KM-plotters on lung cancer patients. The ROC curve was used to assess its diagnostic value. Correlation analysis of ZBTB46 and clinical characteristics, containing T-stage, N stage, sex, smoker, and number of packs smoked per year, was performed with *R* 3.6.3 using the ggplot2 package. TCGA clinical data were used in the above analysis.

### Analysis of DEGs for lung cancer between ZBTB46-high and ZBTB46-low groups and gene set enrichment analysis (GSEA)

In the TCGA dataset (*n* = 1145), ZBTB46 expression was stratified into low/high groups using the median cutoff. Differential expression analysis identified DEGs with |logFC| > 1.5 and FDR < 0.05. GSEA was conducted using the ClusterProfiler *R* package against MSigDB, with significant enrichment defined as FDR (q-value) < 0.25 and adjusted *p* < 0.05.

### Enrichment and network analysis

The Metascape platform was used for functional annotation of DEGs, integrating GO, UniProt, Protein Atlas, and Canonical Pathways. Enrichment analysis via hypergeometric test with Benjamini-Hochberg correction identified significant terms, which were clustered into non-redundant groups based on Kappa-statistical similarity (≥ 0.3). PPI network analysis was performed using the STRING database, followed by the MCODE algorithm in Cytoscape to identify densely connected subnetworks. Biological interpretation was derived via GO enrichment analysis of network components.

### Correlation analysis of immune cell infiltration

Several methods were used for calculating immune infiltration scores based on gene expression data, including TIMER2, ssGSEA, and TISIDB. Immune infiltrate analysis using TIMER2 is a comprehensive resource. The present study correlated ZBTB46 expression in lung cancer with the abundance of immune infiltrates, including B cells, CD4+ T-cells, CD8+ T-cells, neutrophils, macrophages, and dendritic cells. Through single-sample Gene set Enrichment Analysis (ssGSEA), the relative abundance of immune populations was determined across all patients by RNA-seq data obtained from The Cancer Genome Atlas (TCGA).

### Analysis of the correlation between ZBTB46 and immune checkpoints

Spearman’s coefficient assessed gene expression correlations between ZBTB46 and immunoinhibitory genes. Lung cancer RNA-sequencing data were downloaded from the TCGA data portal. Validating these findings, the authors compared gene expression levels with immunoinhibitory gene expression from TIMER2.

### Statistical analysis

The statistical analyses were performed using the *R* software (version 3.6.3) and automatically calculated by the above-mentioned online database. A significant difference was declared at *p* < 0.05. Experimental data were analyzed using GraphPad Prism 9.0.

## Results

### Identification of differentially expressed circRNAs between lung cancer tissues versus paraneoplastic tissues

**Fig. 1:**
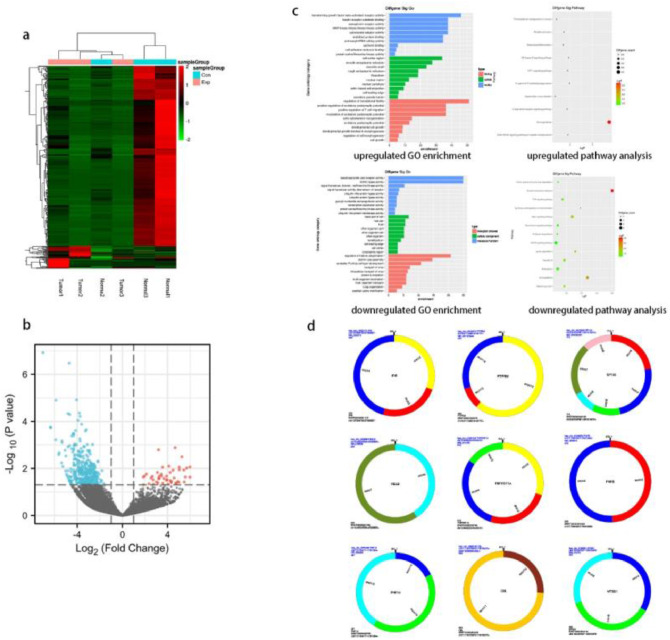
**DEcircRNAs between lung cancer tissues and paracancerous tissues and their functional analysis, structural patterns of candidate circRNAs.** (a) The heat map illustrates the differential circRNA expression (fold change ≥ 1.2 and *p* < 0.05) across lung cancer and adjacent normal lung tissues. (b) Volcano plot shows the different expression of circRNAs. (c) GO enrichment (up) and pathway analysis (down) of upregulated (left) and downregulated (right) circRNAs. (d) Structural patterns of the nine circRNAs. Different colors in the circular structure of the circRNA represent the position of the exon.

The expression of circRNAs in cancer tissues of the lung versus paraneoplastic tissues from three lung cancer patients was compared by high-throughput sequencing technology. There were 80316 circRNA transcripts identified in all samples, of which 411 differentially expressed genes were found, with an absolute log2-fold change greater than 1.2, *p*-value < 0.05. More specifically, cancer tissues showed an upregulation of 46 circRNAs and an upregulation of 365 circRNAs as compared to paraneoplastic tissues. To visualize the differential expression of circRNA between lung cancer tissues and cancer-adjacent tissue, the authors constructed a heat map (Fig. 1a) and a volcano plot (Fig. 1b). Circular RNA expression was considered distinct in lung cancer tissue versus cancer-adjacent tissues. Fig. 1d shows the visual graphical annotation of circRNA. hsa_circ_0002872 is a circular RNA molecule composed of 444 bases, which is formed by exons 12 and 14 of the parental gene. Exons 12 and 14 are closed into a ring, forming a reverse splicing site.

### GO enrichment and KEGG pathway analysis

To characterize the potential roles of circRNAs, parental gene annotations were analyzed across biological processes, cellular components, molecular functions, and pathways. Statistical significance was defined as *p* < 0.05 for correlation analysis (Fig. 1c). Cellular components affected by lung cancer mainly include the cell cortex region, smooth endoplasmic reticulum, dendritic shaft, basal part of the cell, and host cell. For biological processes, lung cancer may affect transforming growth factor beta-activated receptor activity, insulin receptor substrate binding, semaphorin receptor activity, MAP kinase activity, cytoskeletal adaptor activity, misfolded protein binding, lysophosphatidic acid receptor activity, SUMO ligase activity, ubiquitin-protein ligase activity, and transcription coactivator activity. Molecular functions, including regulation of translational fidelity, positive regulation of T cell migration, cell growth, regulation of histone ubiquitination, and protein sumoylation, were the main targets of lung cancer. Pathways including Transcriptional misregulation in cancer, Platelet activation, NF-kappa-B signaling pathway, HIF-1 signaling pathway, Fc gamma R-mediated phagocytosis, TNF signaling pathway, Rap1 signaling pathway, MAPK signaling pathway, Adherens junction, etc., are regulated by lung cancer, according to KEGG pathway analysis.

### Prediction of the most potential circRNA/miRNA/mRNA axis

**Fig. 2:**
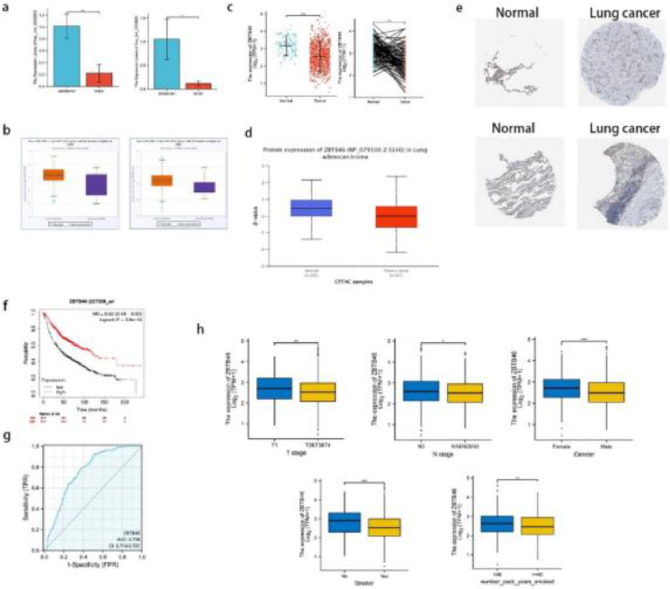
**qRT-PCR verified that candidate circRNAs are lowly expressed in lung cancer; lower ZBTB46 expression is associated with a better prognosis in lung cancer patients.** (a) The levels of hsa_circ_0002872 and hsa_circ_0135681 expression in 15 lung cancer tissues vs adjacent tissues were assessed using qRT‐PCR. (b) The expressions of hsa-miR-29b-1–5p in LUAD and LUSC patients were analyzed from the starBase of ENCORI. (c) A significant increase in ZBTB46 expression was observed in lung cancer tissues compared to normal tissue, in both paired and unpaired analyses. (d) An examination of the protein expression of ZBTB46 in tissues from LUADs and normal lungs (CPTAC database). In patients with LUAD, ZBTB46 protein expression was significantly decreased from the CPTAC database. (e) Lung cancer samples were found to contain a lower level of ZBTB46 protein than normal tissues (HPA database). (f) Kaplan-Meier survival curves demonstrated a high ZBTB46 level in lung cancer patients with a good prognosis. (g) According to ROC analysis, ZBTB46 was able to distinguish tumors from normal tissue accurately. The AUC value was 0.758. (h) The association between ZBTB46 and clinical manifestation in lung cancer. There was an association between higher ZBTB46 expression and lower T-stage, lower N-stage, female, no smoker, and number of packs smoked per year < 40. * Indicates *p* < 0.05, ** Indicates *p* < 0.01, ***Indicates *p* < 0.001.

Based on GO and KEGG analysis of the circRNA parental gene, the authors identified 9 major candidate circRNAs associated with the development and progression of lung cancer from among 411 DEcircRNAs. The basic structure patterns of 9 candidate circRNAs are presented in Fig. 1d. Validate the expression of these 9 candidate circRNAs in 15 pairs of lung cancer tissues and their matched normal tissues by qRT-PCR. Comparing lung cancer tissues with adjacent tissues, hsa_circ_0002872 and hsa_circ_0135681 show reduced expression. The results of qRT-PCR verification were consistent with the sequencing results (Fig. 2a). Analysis of the circAtlas website showed that hsa_circ_0002872 targets 2 miRNAs and hsa_circ_0135681 targets 5 miRNAs. After analyzing multiple online websites to find the target mRNAs of the above miRNAs, the authors found that hsa-miR-29b-2–5p and hsa-miR-29b-1–5p target 11 mRNAs together (Table 2). Using public clinical data, the Kaplan-Meier survival analysis found that ZBTB46, one of these 11 genes, is associated with a higher survival rate among lung cancer patients than those with low levels, implying that ZBTB46 expression may be associated with lung cancer protection. In lung cancer tissue, ZBTB46, like hsa_circ_0002872, has a lower expression level than in normal lung tissue. Meanwhile, there is a body of emerging literature that implicates ZBTB46 abnormal expression in malignancies. Therefore, based on ceRNA theory, the authors selected hsa-miR-29b-1–5p, which is highly expressed in lung cancer (Fig. 2b), and predicted the most potential downstream circRNA axis: hsa_circ_0002872/hsa-miR-29b-1–5p/ZBTB46.

**Table 2:** 11 common elements in hsa-miR-29b-1-5p and hsa-miR-29b-2-5p.

Gene	Expression of genes in lung cancer (Gepia2)	Kaplan-Meier Plotter curve (p-values)	ROC-plot AUC value (TCGA)
CREBZF	Up	0.0038	0.745
CENPM	Up	2.4e-11	0.932
GPR155	No differential expression	0.66	0.512
CORO2A	Up	6e-06	0.670
CRTC3	Down	1.6e-05	0.735
KITLG	Down	0.72	0.659
SPOUT1	Up	-	0.783
KCNK5	No differential expression	3.8e-12	0.529
PLEKHM1	Down	0.0033	0.673
ZBTB46	Down	3.9e-14	0.758
RPA1	Up	0.015	0.687

### ZBTB46 expression in lung cancer and correlation analysis of prognosis

In the TCGA database, ZBTB46 was measured for its mRNA expression pattern. According to Fig. 2c, lung cancer tissues expressed significantly less ZBTB46 mRNA than normal samples. CPTAC and HPA were used to explore the protein expression of ZBTB46 in lung cancer following mRNA analysis. According to CPTAC, lung cancer tissues expressed less ZBTB46 protein than normal tissues in Fig. 2d. As shown in Fig. 2e, lung cancer tissues expressed low levels of ZBTB46 proteins, as determined by HPA, compared with normal tissues. Lung cancer tissues expressed low levels of ZBTB46 protein, whereas they were medium levels in normal lung tissues. In conclusion, the authors found that patients with lung cancer had significantly low levels of ZBTB46 protein expression. A high ZBTB46 level is associated with a good prognosis using the Kaplan-Meier survival curve as a guide (Fig. 2f). Under the ROC curve, the Area Under the Curve (AUC) was 0.758 (Confidence Interval: 0.719‒0.797) (Fig. 2*g*). This result suggests that ZBTB46 can distinguish between normal and tumor tissues. Statistically, there was a difference in the correlation analysis between ZBTB46 and T-stage (*p* < 0.01), N-stage (*p* < 0.05), gender (*p* < 0.001), smoker (*p* < 0.001), and number of packs smoked per year (*p* < 0.01) (Fig. 2h).

### Differentially expressed genes analysis and GSEA analysis

**Fig. 3:**
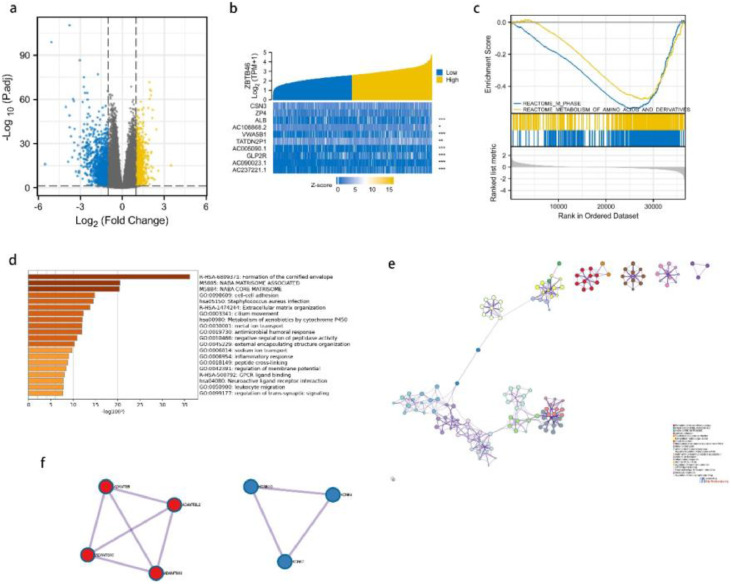
**ZBTB46 and its neighboring genes' enrichment analysis in the TCGA database, Gene Set Enrichment Analysis (GSEA), and OC (Metascape).** (a) It is a volcano plot representing the genetic differential expression map of lung cancer in TCGA. The X-axis represents log2(FC), and larger absolute values indicate larger fold changes. Y-axis represents the corrected -log10 (*p*-value), with higher values indicating a significant difference. (b) A heatmap is shown. Yellow indicates high expression, while blue indicates low expression. (c) Gene Set Enrichment Analysis (GSEA) enrichment maps. According to the GSEA results, m phase and metabolism of amino acids and derivatives were enriched mainly in ZBTB46-related lung cancer. (d) The enrichment analysis of ZBTB46 in Metascape. All statistically enriched terms (including GO, KEGG pathway, etc.) were identified; the top 20 of them are shown here. (e) An interaction network between Proteins and Proteins (PPI). (f) Two major components of MCODE constitute the PPI network.

The analysis of Differentially Expressed Genes (DEGs) was conducted using TCGA cohort data. Depending on the expression level of ZBTB46, lung cancer patients were divided into two groups: the high-expression group and the low-expression group. As a result of screening, 2049 genes were found to be differentially expressed. According to Fig. 3a, 989 genes were expressed highly, and 1060 genes were expressed lowly. A heatmap of gene expression was created to obtain an overview of the top 10 most significant genes in expression in lung cancer (Fig. 3b). To gain a deeper understanding of the biological pathways involved in lung cancer with differing levels of ZBTB46 expression, GSEA analysis was performed. To identify critical signaling pathways involved in lung cancer, GSEA was conducted between datasets high and low in ZBTB46 expression levels. In the MSigDB Collection, these pathways were significantly enriched (Fig. 3c). GSEA results showed that M_PHASE and METABOLISM_OF_AMINO_ACIDS_AND_DERIVATIVES were closely correlated with ZBTB46 expression by GSEA analysis.

### ZBTB46 expression phenotype-enriched gene sets and protein-protein interaction

A comparison of GO and KEGG in Metascape was used to predict ZBTB46′s functions (Fig. 3d). Cell-cell adhesion, cilium movement, metal ion transport, antimicrobial humoral response, negative regulation of peptidase activity, external encapsulating structure organization, sodium ion transport, inflammation response, leukocyte migration, and trans-synaptic signaling were among the top 20 GO enrichment items. According to the KEGG pathway analysis, the formation of the cornified envelope, extracellular matrix organization, and metabolism of xenobiotics were found to be the pathways that had the greatest significance. As an additional step towards better understanding ZBTB46′s potential biological function, the authors conducted a metascape analysis of protein-protein interactions. Fig. 3e depicts an overview of the protein-protein interaction network as well as the MCODE components that are identified in the gene listing. Following the pathway as well as process enrichment analyses for each component of the MCODE, the results are consistent with GO and KEGG. ZBTB46 is regulated in lung cancer by multiple genes interacting functionally. The Molecular Complexity Detection (MCODE) algorithm was employed to identify a densely connected network, resulting in 7 hub genes, comprising ADAMTSL2, ADAMTS8, ADAMTS10, ADAMTS16, KCNK4, KCNK7, and KCNK10, which can be seen in Fig. 3f. A majority of these hub genes are members of the ADAMTS and KCNK families.

### ZBTB46 positively correlates with immune cell infiltration

**Fig. 4:**
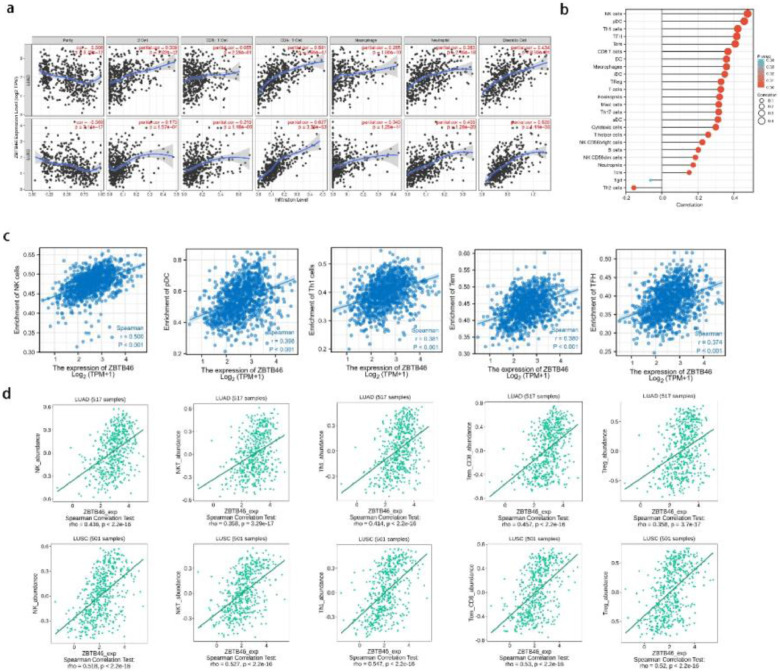
**Correlation between ZBTB46 expression and lung cancer immune infiltration level.** (a) Significantly positive correlations exist between ZBTB46 expression and immune cells, including B-cells, CD8+ T-cells, CD4+ T-cells, macrophages, neutrophils, and dendritic cells in LUAD and LUSC. (b) Lollipop diagram of twenty-four immune infiltration cells from TCGA lung cancer tissues. (c) Study of the correlation of ZBTB46 expression level with infiltrating immune cells using the TCGA database. The expression of ZBTB46 exhibited a significant positive correlation with infiltrating levels of NK cells, pDC, Th1 cells, Tem, and TFH. (d) Correlation between the expression of ZBTB46 and the degree of immune infiltration (including NK cells, NKT cells, Th1 cells, Tem_CD8 and TReg) from the TISIDB website.

ZBTB46 was previously considered a conventional Dendritic Cell (cDC) marker, but now researchers have identified a new subpopulation of immune cells expressing ZBTB46, revealing a new pathway mediated by ZBTB46 that regulates the ILC3 immune response. We analyzed TIMER data to determine whether ZBTB46 expression levels correlated with immune cell infiltration levels. As presented in Fig. 4a, it was observed that ZBTB46 expression correlated significantly positively with all types of immune cells analyzed, including B-cells, CD8+ T-cells, CD4+ T-cells, macrophages, neutrophils, and dendritic cells in lung cancer. Immune ssGSEA using lung cancer TCGA data confirmed that 5 immune infiltrating cells positively associated with ZBTB46 were NK cells (*r* = 0.500, *p* < 0.001), pDC (*r* = 0.398, *p* < 0.001), Th1 cells (*r* = 0.381, *p* < 0.001), Tem (*r* = 0.380, *p* < 0.001) and TFH (*r* = 0.374, *p* < 0.001) (Fig. 4b‒c). Notably, in addition to Tgd and Th2 cells, the other 22 immune infiltrating cells examined were all positively correlated with ZBTB46. To further verify the above conclusions, lung cancer immune infiltration levels and ZBTB46 expression were then correlated using TISIDB. A positive correlation was observed between ZBTB46 expression and NK cells, NKT cells, Th1 cells, Tem_CD8, and TReg (Fig. 4d). Overall, ZBTB46 may alter immune responses in the tumor microenvironment through its effect on immune cells.

### ZBTB46 positively correlates with immune checkpoints in lung cancer

**Fig. 5:**
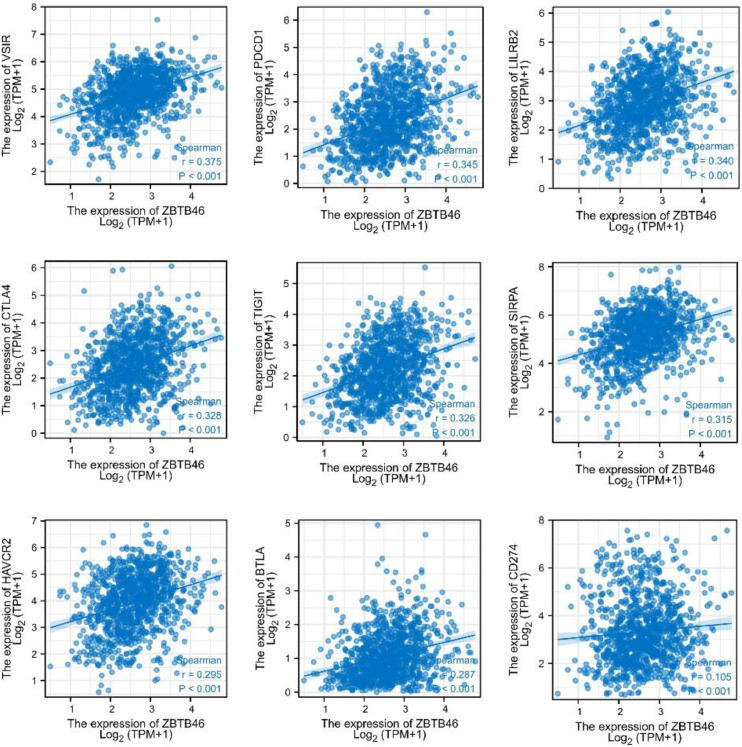
**The positive correlation between ZBTB46 and immune checkpoint molecules** (VSIR, PD1, LILRB2, CTLA-4, TIGIT, SIRPA, HAVCR2, BTLA, and PD-L1) in TCGA lung cancer samples.

Immune checkpoints are crucial to the evasion of the immune system in tumors. A relationship between Immune checkpoints and ZBTB46 was examined considering ZBTB46′s potential tumor suppressor role in lung cancer. As suggested in Fig. 5, ZBTB46 expression was significantly positively correlated with VSIR (Spearman’s *r* = 0.375, *p* < 0.001), PD1 (Spearman’s *r* = 0.345, *p* < 0.001), LILRB2 (Spearman’s *r* = 0.340, *p* < 0.001), CTLA-4 (Spearman’s *r* = 0.328, *p* < 0.001), TIGIT (Spearman’s *r* = 0.326, *p* < 0.001), SIRPA (Spearman’s *r* = 0.315, *p* < 0.001), HAVCR2 (Spearman’s *r* = 0.295, *p* < 0.001), BTLA (Spearman’s *r* = 0.287, *p* < 0.001) and PD-L1 (Spearman’s *r* = 0.105, *p* < 0.001) in lung cancer. It appears that ZBTB46 can inhibit lung tumor development via tumor immune escape.

## Discussion

Epigenetic mechanisms intricately regulate cancer progression, including lung cancer, with circRNAs emerging as critical modulators in transcriptional and post-transcriptional regulation. Characterized by their covalently closed structures, these non-coding RNAs have been implicated in tumorigenesis and metastasis, positioning them as promising therapeutic targets.

ZBTB46 (BTBD4), a BTB-ZF transcription factor, is selectively expressed in conventional Dendritic Cells (cDCs),^13^ absent in monocytes/macrophages and other myeloid/lymphoid lineages.^14^ Endothelial Cells (ECs) exhibit minimal ZBTB46 expression compared to cDCs.^20^ Functionally, ZBTB46 potently inhibits EC proliferation in *vitro*/in *vivo* by inducing G0-G1 arrest and downregulating Cyclin-Dependent Kinases (CDKs).^21^^,^^22^ A seminal study revealed that ZBTB46+ ILC3s regulate intestinal inflammation by attenuating pro-inflammatory responses, inhibiting OX40L-dependent Th17 expansion, and producing IL-22. Depletion of ZBTB46+ ILC3s exacerbates enteropathogenic infections and inflammation in murine models.^23^ A hallmark cancer-predisposing factor is chronic inflammation, which is associated with reduced “beneficial” flora.^24^ While ZBTB46 promotes tumorigenesis in neuroendocrine prostate cancer,^16^ its role in lung cancer remains underexplored. Thus, elucidating the therapeutic and prognostic relevance of ZBTB46 in lung cancer is imperative.

In this study, RNA-seq was conducted on three paired healthy and lung cancer serum samples to identify circRNAs. Differential expression analysis identified 411 circRNAs significantly altered in lung cancer, implicated in tumorigenesis. Findings from functional enrichment analysis indicated that the parental genes of annotated circRNAs were associated with TNF, Rap1, and MAPK signaling pathways,^25^ which have been linked to lung cancer development. Based on ceRNA theory, we predicted the most promising downstream circRNA axis: hsa_circ_0002872/hsa-miR-29b-1–5p/ZBTB46. Bioinformatics approaches were then applied to the TCGA database and multiple online analysis platforms, compared with adjacent normal tissues, lung cancer tissues exhibited lower expression levels of ZBTB46 mRNA and protein. Moreover, ZBTB46 expression was significantly associated with clinicopathological parameters (T/N stage, gender, smoking status/pack-years), indicating its involvement in lung cancer pathogenesis. Intriguingly, a significant correlation was observed between ZBTB46 expression and overall survival, with the high-expression group experiencing significantly higher survival rates than the low-expression group. Analysis of the relationship between ZBTB46 and immune infiltrating cells and immune checkpoint inhibitor molecules suggested that ZBTB46 correlated positively with the expression of most of the immune infiltrating cells and immune checkpoint inhibitors. In conclusion, hsa_circ_0002872/hsa-miR-29b-1–5p/ZBTB46 is an axis that may influence lung cancer progression. ZBTB46 is a key molecule that inhibits lung cancer progression and is considered a significant prognostic marker for lung cancer patients. Gene Set Enrichment Analysis (GSEA) revealed that ZBTB46 expression was closely associated with the M-phase and amino acid metabolism. The target mRNA was also found to be involved in various biological processes and metabolic pathways, such as extracellular matrix organization, ciliary movement, and leukocyte migration, which are crucial for maintaining tumor cell energy homeostasis and significantly influence tumor cell survival, death, differentiation, proliferation, and inflammation.^26^^,^^27^

Deciphering the mechanisms of immune responses to tumor growth and immunotherapy hinges on understanding the cancer immune microenvironment. Immune cells within this milieu regulate cancer progression and represent critical therapeutic targets.^28^ Multiple studies have linked immune cell infiltration to lung cancer survival.^29^, ^30^, ^31^ This study revealed that ZBTB46 expression in LUAD and LUSC is significantly correlated with immune cell infiltration, including B-cells, CD8+ T-cells, CD4+ T-cells, macrophages, neutrophils, Dendritic Cells (DCs), NK cells, pDC, Th1 cells, Tem, TFH, NKT cells, Tem_CD8, and TReg.

T-cells constitute the majority of immune cells in the NSCLC tumor microenvironment, followed by B-cells, macrophages, DCs, and natural killer cells.^32^ Divergent T-cell responses in lung cancer are associated with differential infiltration of classical Dendritic Cells (cDCs),^33^ and low effector T-cell infiltration correlates with poor NSCLC prognosis.^34^ cDC lineages selectively express transcription factor ZBTB46 (BTBD4).^35^ A prior study demonstrated that among 13 tumor-infiltrating immune cell populations, substantial infiltration of 12 cell types correlated with favorable prognosis, whereas high Th2 cell infiltration was associated with poor prognosis specifically in LUAD patients.^36^ As a transcriptional repressor, ZBTB46 regulates tumor angiogenesis and modulates vascular endothelial cell function. Additionally, ZBTB46 remodels immune cell composition and enhances anti-tumor immune effector functions in the tumor microenvironment, thereby influencing cancer prognosis.^37^ Consistent with these findings, the present study showed high ZBTB46 expression predicted favorable outcomes in lung cancer, with significant negative correlation with Th2 cell infiltration. These results underscore the importance of ZBTB46 in shaping the tumor immune microenvironment, warranting further investigation into its precise role in lung cancer progression.

The sustained antitumor efficacy of Immune Checkpoint Inhibitors (ICIs) relies on tumor cells' high-level expression of immune checkpoints.^38^ Key immune checkpoints, including CTLA-4, PD-1, and PD-L1, are frequently studied in this context. This study revealed that tumors with high ZBTB46 expression also exhibit elevated CTLA-4, PD-1, and PD-L1 levels, which correlated with favorable patient outcomes. These findings suggest ZBTB46 may modulate immune checkpoint pathways, raising the question: Does this regulatory mechanism predict responsiveness to immune checkpoint blockade? We hypothesize that ZBTB46-mediated immune checkpoint activation could enhance therapeutic sensitivity to ICIs.

This study has several limitations. First, it represents a preliminary investigation into ZBTB46′s role in lung cancer, requiring additional experiments to validate predicted molecular mechanisms. Second, experimental verification of circRNA-miRNA/mRNA interactions via dual-luciferase reporter and RNA pull-down assays is necessary. Third, mechanistic evidence linking ZBTB46 to immune infiltration and tumor-immune interactions remains limited. Although circRNA data were generated, time and methodological constraints precluded in-depth exploration of ZBTB46-circRNA regulatory networks. Future studies should prioritize in *vivo* validation of these findings and integrate additional clinical data from TCGA to strengthen prognostic associations. ZBTB46 was identified as a novel lung cancer biomarker in this study. It contributes to understanding cell cycle regulation and immune infiltration/checkpoint pathways in lung carcinogenesis. Further functional characterization may establish ZBTB46 as a promising diagnostic and therapeutic target for lung cancer.

## Conclusion

Collectively, the present findings identify the hsa_circ_0002872/hsa-miR-29b-1–5p/ZBTB46 axis as a novel regulator of lung cancer progression, with ZBTB46 serving as a potential prognostic marker and therapeutic target.

### Ethics approval and consent to partipate

The authors are accountable for all aspects of the work, including ensuring that any questions related to the accuracy or integrity of any part of the work have been appropriately investigated and resolved. All participants signed an informed consent form, and this study was approved by the institutional Ethics Board of Jinling Hospital (Approval No.2020DZGZRZX-096). All procedures performed in this study involving human participants and genes were by the Declaration of Helsinki (as revised in 2013).

## Authors’ contribution

FFC and XFW designed the research study. XFW and YXS performed the research. YXS is revising it critically for important intellectual content. When starting to revise the manuscript, DCZ contributed extremely crucial revision suggestions. XQL provided help and advice on conception, and HXW analyzed the data. XHW, YXS, HHL, and YZ wrote the manuscript. All authors contributed to editorial changes in the manuscript. All authors read and approved the final manuscript.

## Funding

This work was supported by the following grants: 10.13039/501100001809National Natural Science Foundation of China (Grant No. 82002233),Clinical Diagnosis and Treatment New Technology Project (Grant No. 22LCZLXJS58), Basic Research Project of Jinling Hospital (Grant No. 2023JCYJYB105), New Technology of Clinical Diagnosis and Treatment Project of Jinling Hospital (Grant No. 2023LCZLXB044), Key Project of 10.13039/501100010227Nanjing Medical Science and Technology Development Program (Grant No. ZKX23037), Key Project of Talent Support Program of Nanjing Second Hospital (Grant No. RCZD202302), General Project of Special Research Fund for Post-marketing Clinical Research of Innovative Drugs of the 10.13039/501100004572National Health Commission (Grant No. WKZX2024CX101211).

## Declaration of competing interest

The authors declare no conflicts of interest.
